# Supplementary material for: Consumption of non-sugar sweeteners by Brazilian adolescents and adults in 2017–2018: Socioeconomic distribution and food sources
Source: PLoS One. 2025 Nov 17;20(11):e0334091. doi: 10.1371/journal.pone.0334091 (PMC12622844; doi:10.1371/journal.pone.0334091)
Supplement: S1 File — (DOCX) [file pone.0334091.s001.docx]

[**Supporting information**](https://www.ncbi.nlm.nih.gov/pmc/articles/PMC8490357/) **1**

**Preparation of the Food Additives Table to estimate the non-sugar sweeteners present in foods reported by the 2017-2018 Household Budget Survey**

**Data source**

The process for identifying non-sugar sweeteners in foods consumed by Brazilians was carried out using two data sources: A dataset with brands of food sold in Brazil - Euromonitor (2017); and a dataset of labels of food sold in Brazil - developed by the Brazilian Institute for Consumer Protection (IDEC/2017) in partnership with the Center for Epidemiological Research in Nutrition and Health at the University of São Paulo (NUPENS/USP).

Euromonitor International is a global consumer and market intelligence company. The institution provides databases, reports and insights to its clients in order to anticipate trends in industries, the economy and consumption.

The data source used in this study provided marketing information from the food retail and food service sector. The variables in this source were: country of origin of the data, food categories and subcategories, product brands and the respective company in which the company is inserted, the year/period of the information and the percentage of food sales by product brand.

The food label dataset was developed by the Brazilian Institute for Consumer Protection (IDEC) in partnership with the Center for Epidemiological Research in Nutrition and Health at the University of São Paulo (NUPENS/USP) and provided the list of ingredients for foods with labels. The collection took place between April and July 2017 and was carried out by trained evaluators, using a photographic method to record the labels, with images of all sides of the food and beverage packaging. Taking photographs of packaged food products in supermarkets is considered an effective way of collecting information contained on food labels, previously assessed through research or direct purchase of the products. Photographic methods are less expensive and more viable than conducting paper research or purchasing food products, which have restrictions on hard copies or storage space (KANTER, REYES AND CORVALAN, 2017).

All packaged foods and beverages available for sale in the selected supermarkets were included in the study, totaling around 14.000 products photographed. After deleting duplicate products, 12.956 products were retained in the data source. Products available in different sized packages, with multiple packages and varied items, bottled water, and products without nutritional information available were also excluded. Finally, 11.434 foods made up the data source. More details on data collection are available in Duran et al. (2020). Only foods and beverages that had ingredient list data were included, since natural foods such as vegetables, fruits, fresh herbs (parsley, basil), legumes (chickpeas, lentils, beans), cereals (rice, corn, quinoa, tapioca starch), seeds (chia, sesame, flaxseed), and coffee do not have an ingredient list. Therefore, in the end, a total of 9.856 items were evaluated.

**Identification of food additives**

The process to identify food additives for food items in the 2017-2017 POF took place in three phases:

Phase 1 - For POF food items that featured the product brand (e.g. Tang Soft Drink), the corresponding products and their respective list of ingredients were selected directly from the food label dataset (IDEC & NUPENS) (e.g. Tang, Orange, 25G, B - sugar, maltodextrin, dehydrated orange juice, vitamin C, zinc sulfate, vitamin D, citric acid acidulant, non-sugar sweeteners: aspartame, sodium cyclamate, acesulfame potassium and sodium saccharin, acidity regulator potassium citrate, anti-caking agent tricalcium phosphate, thickeners: guar and xanthan gums, flavoring (contains soy derivative), dyes: titanium dioxide, tartrazine and sunset yellow FCF and sparkling quillaia extract.)

Phase 2 - For food items in the consumption dataset (POF 2017-2017) that did not have a brand name, the Euromonitor database was used to identify the most consumed brand in Brazil, for each food or food group. This brand was used as a reference to identify the list of food additives.

Using the information on each food item (name and brand), the products were identified with the respective description in the food label dataset (IDEC & NUPENS). In the food brand bank (Euromonitor), information on the three most consumed brands was selected for each food item, that is, if the product with the first most consumed brand was not found, the second was chosen, followed by the third brand. For example, the item from the consumption bank “green tea”, plus the information on the most consumed brand of industrialized tea from the bank with food brands “Leão”, resulted in the identifying the product from the label bank “Chá Verde Limão, 1L, T”.

Phase 3 - For food items from the consumption bank (POF 2017-2017) that were not identified in the food label bank (IDEC & NUPENS), according to the bank with food brands (Euromonitor), the following criteria were established:

- If there is only one product in the food label bank (IDEC & NUPENS) for the food item in the consumption bank (POF 2017-2017) - Select the product;
- If there are 2 products in the food label bank (IDEC & NUPENS) for the food item in the consumption bank (POF 2017-2017) - Select the product that has fewer additive functions in the ingredients list;
- If there are 3 or more products in the food label bank (IDEC & NUPENS) for the food item in the consumption bank (POF 2017-2017) - add up the number of additive functions for each product. Then, identify the products with the “mode” among the sums - if the additive function profile is the same, select any brand - if the function profile is different, select the one with the most similar profile among the selected products. For example, the additives trend for whole wheat toast was four technological functions. Three products had four additives, but products number 1 and 3 had the same additives (flavoring, emulsifier, preservative and flour improver), while product number two had (emulsifier, colorant, thickener and flour improver). Thus, product number 1 or 3 was selected to represent the additive profile of whole wheat toast.;
- If there are 3 or more products in the food label bank (IDEC & NUPENS) for the food item in the consumption bank (POF 2017-2017) and not present the "mode" after the sum of the additive functions - select the product that has the least additive functions. For example, for the Murfin product, three products were found; however, product 1 presented 7 additive functions, product 2 had 4 functions and product 3 presented 6 functions. Therefore, product 2 was selected, as it presented the smallest number of functions.;
- If the product does not exist in the food label bank (IDEC & NUPENS) for the food item in the consumption bank (POF 2017-2017) - a) identify whether the food item is diet, light or zero and then check if there is another product with any of that information. For example, if “diet cola” was not found, check if “light or zero cola” exists. Select a product with similar specifications. b) For very specific foods, select another similar product taking into account the similarity of additive functions; c**)** As a last resort, disaggregate the food and identify the food additives based on the ingredients.
