# Supplementary material for: Consumption of non-sugar sweeteners by Brazilian adolescents and adults in 2017–2018: Socioeconomic distribution and food sources
Source: PLoS One. 2025 Nov 17;20(11):e0334091. doi: 10.1371/journal.pone.0334091 (PMC12622844; doi:10.1371/journal.pone.0334091)
Supplement: S2 Table — Brazil, 2017–2018. Note: The non-suger sweeteners Steviol (INS 960), Isomalt (INS 953), Lactitol (INS 966), Erythritol (INS 968), Thaumatin (INS 957), Xylitol (INS), Manitol (421) and Neotame (INS 961) had a very low frequency (≤ 15), so they were not included in the analyses and in the table. 1International Numbering System for Food Additives (INS). (DOCX) [file pone.0334091.s002.docx]

Supporting Information 2

| **Table 1. Total frequency of non-sugar sweeteners consumed in foods and prevalence (%) of consumers of non-sugar sweeteners in foods, according to compounds classified as non-sugar sweeteners, in individuals aged ≥ 10 years. Brazil, 2017-2018** | | | | | | | | | | |
| --- | --- | --- | --- | --- | --- | --- | --- | --- | --- | --- |
|  |  |  |  | **Prevalence of concomitant consumption of non-sugar sweeteners (%)** | | | | | | |
| **Non-sugar sweeteners** | **INS^1^** | **Frequency** | **Prevalence (%)** | Acesulfame K | Sucralose | Aspartame | Saccharins | Cyclamates | Sorbitol | Maltitol |
| Acesulfame K | 950 | 8047 | 19.8 | 7.6 |  |  |  |  |  |  |
| Sucralose | 955 | 6319 | 15.8 | 15.0 | 6.0 |  |  |  |  |  |
| Aspartame | 951 | 2354 | 6.0 | 5.9 | 0.0 | 1.3 |  |  |  |  |
| Saccharins | 954 | 2283 | 5.5 | 5.5 | 1.0 | 5.4 | 1.2 |  |  |  |
| Cyclamates | 952 | 2245 | 5.5 | 5.5 | 1.0 | 5.5 | 5.4 | 1.2 |  |  |
| Sorbitol | 420 | 325 | 0.8 | 0.3 | 0.3 | 0.0 | 0.0 | 0.0 | 0.0 |  |
| Maltitol | 965 | 60 | 0.2 | 0.0 | 0.2 | 0.0 | 0.0 | 0.0 | 0.0 | 0.0 |
| Note: The non-suger sweeteners Steviol (INS 960), Isomalt (INS 953), Lactitol (INS 966), Erythritol (INS 968), Thaumatin (INS 957), Xylitol (INS), Manitol (421) and Neotame (INS 961) had a very low frequency (≤ 15), so they were not included in the analyses and in the table.  ^1^International Numbering System for Food Additives (INS) | | | | | | | | | | |
